# The Differential Complement, Fc and Chemokine Receptor Expression of B Cells in IgG4-Related Pancreatobiliary Disease and Primary Sclerosing Cholangitis and Its Relevance for Targeting B Cell Pathways in Disease

**DOI:** 10.3390/biomedicines12122839

**Published:** 2024-12-13

**Authors:** Tamsin Cargill, Eleanor Barnes, Theo Rispens, Emma L. Culver

**Affiliations:** 1 Peter Medawar Building for Pathogen Research, Nuffield Department of Medicine, University of Oxford, South Parks Road, Oxford OX1 3SY, UK; 2 Translational Gastroenterology and Liver Unit, Nuffield Department of Medicine, University of Oxford, John Radcliffe Hospital, Oxford OX3 9DU, UK; 3 Oxford NIHR Biomedical Research Centre, University of Oxford, Oxford OX3 9DU, UK; 4 Sanquin, Division Research and Landsteiner Laboratory, Academic Medical Center, University of Amsterdam, Plesmanlaan 125, 1066 CX Amsterdam, The Netherlands

**Keywords:** IgG4, IgG4-related disease, primary sclerosing cholangitis, B cells, Fc receptor, complement

## Abstract

**Background:** Immune-mediated liver and biliary conditions, such as IgG4-related pancreatobiliary disease (IgG4-PB) and a subset of primary sclerosing cholangitis (PSC- high(h)IgG4), exhibit increased IgG4 levels in the blood. The relative expression of IgG4+ and IgG1+ B cells in the blood and the expression of complement and Fc receptors on these IgG1+ and IgG4+ B cells in IgG4-PB and PSC have not been previously described. We hypothesised that the patterns of expression of these cells and their receptors would differ, are relevant to disease pathogenesis and may represent therapeutic targets. **Methods:** CD19+ B cells were sorted from blood collected from patients with IgG4-PB, PSC-high(h)IgG4 and healthy volunteers. Cells were stained with fluorescent labelled antibodies specific to IgG1, IgG4, complement receptors (CR1 and CR2), Fc receptors (FcεRII and FcγRIIb) and chemokine receptors (CXCR3, CXCR4, CXCR5) and were analysed by flow cytometry. **Findings:** IgG4-PB, compared to healthy volunteers, showed decreased CR2 expression on IgG1+ B cells (MFI 416 (275–552) vs. 865 (515–3631), *p* = 0.04) and IgG4+ B cells (MFI 337 (231–353) vs. 571 (398–2521), *p* = 0.03). IgG4-PB, compared to healthy volunteers, showed increased FcεRII expression on IgG4+ B cells (MFI 296 (225–617) vs. 100 (92–138), *p* = 0.0145) and decreased FcγRIIb expression on IgG1+ B cells (134 (72–161) vs. 234 (175–291), *p* = 0.0262). FcγRIIb expression was also decreased in IgG1+ B cells in patients with PSC-hIgG4 compared to healthy volunteers. **Conclusions:** This exploratory study indicates that in IgG4-PB, B cells have decreased CR2 and FcγRIIb expression and increased FcεRII expression, suggesting altered sensitivity to complement, IgG-mediated inhibition and sensitisation by IgE, which may promote the relative expansion of IgG4+ B cells in this disease.

## 1. Introduction

The B cell lineage forms an important part of the adaptive immune system, essential for long-term antibody-mediated immunity [1]. The classes of antibody present in humans (IgM, IgD, IgE, IgA and IgG) are defined by their Fc region. IgG responses have four subclasses (IgG1, IgG2, IgG3 and IgG4) and are produced only when B cells have undergone class-switch recombination. In health, IgG4 antibodies constitute only 4% of the circulating IgG pool, with IgG1 being the most common [2]. IgG4 antibodies have distinct properties, including fragment antigen binding (Fab)-arm exchange and differential Fc receptor binding, which renders them poor activators of complement and immune-cell-mediated cytotoxicity [2].

In addition to directly neutralising pathogens, antibodies act through their fragment crystallisable (Fc) region to activate the complement cascade or interact with other immune cells, resulting in cytotoxicity or phagocytosis [3]. These functions are modulated through interactions between complement, Fc and chemokine receptors and their ligands. IgG4+ B cells have been found to have a distinct phenotype compared to IgG1+ B cells, with reduced expression of complement receptors, suggesting they are less sensitive to signalling through complement components [4]. This suggests IgG4+ B cells and IgG4 antibodies play an immunoregulatory rather than pro-inflammatory role in health.

Several immune-mediated conditions are associated with elevated circulating IgG4 levels, raising the possibility that in particular disease states, IgG4+ B cells may be dysregulated. IgG4-related disease (IgG4-RD) is a fibroinflammatory relapsing-remitting condition characterised by raised serum IgG4 levels and lesions in single or multiple organs, most commonly affecting the pancreas and bile ducts (IgG4-related pancreatobiliary disease, IgG4-PB) [5,6]. The histological hallmarks include infiltration of IgG4+ plasma cells, storiform fibrosis, and obliterative phlebitis [7]. IgG4+ B cells are implicated in disease pathogenesis in several ways, including the production of self-reactive IgG4 antibodies, the presentation of antigens to T cells and the promotion of fibrogenesis [8]. Primary sclerosing cholangitis (PSC) is an immune-mediated condition leading to bile duct fibrosis and strictures, mimicking IgG4-PB of the biliary tree [9]. An altered gut microbiome and the loss of tolerance to shared gut–liver antigens have been proposed as a potential mechanism of PSC pathogenesis [10]. B cell involvement is suggested by the association of autoantibodies, such as anti-neutrophil cytoplasmic antibodies (ANCA) [8]. A subset of PSC patients with a raised serum IgG4 (PSC-hIgG4) may have more aggressive diseases than those with normal IgG4 levels, but the reasons for this are not clear [11].

Targeting B cells in autoimmune diseases can induce and maintain clinical disease remission. In IgG4-RD, the depletion of B cells by the monoclonal anti-CD20 antibody, Rituximab, can improve clinical, biochemical, and radiological evidence of active disease [12,13,14,15,16,17,18,19,20]. A recent phase 3 randomised clinical trial investigating Inebilizumab, a monoclonal antibody that depletes CD19 B cells (MITIGATE) in IgG4-RD, has shown Inebilizumab treatment increases the likelihood of complete remission at 1 year [21]. However, a pan-B cell depletion approach to therapy renders a patient vulnerable to infections usually controlled by humoral immunity, so alternative approaches inhibiting B cells such as Obexelimab, a monoclonal antibody that binds to CD19 and FcyIIb (Phase 3 INDIGO), or targeting specific B cell pathways such as Bruton tyrosine kinase inhibitors and B cell activating factor (BAFF) inhibitors are also under investigation [22,23,24]. In PSC, anti-CD20 Rituximab induction has been used to prevent the recurrence of PSC after a liver transplant [25]. A better understanding of B cell molecular pathways in these conditions is necessary to identify novel disease-specific B cell therapeutic targets.

The expression of complement, Fc and chemokine receptors on B cells in IgG4-PB and PSC has not been previously described. We hypothesised that the patterns of expression would differ between IgG1+ and IgG4+ B cells in patients with IgG4-PB and PSC-hIgG4, which might be relevant to disease pathogenies and/or represent therapeutic targets. We demonstrate an altered receptor expression repertoire in IgG4+ and IgG1+ B cells in IgG4-PB and PSC-hIgG4, suggesting an altered sensitivity to complement, chemokine and IgG-mediated inhibition in these diseases.

## 2. Materials and Methods

### 2.1. Study Design

Patients with IgG4-PB or PSC-hIgG4 and healthy volunteers were recruited prospectively from Oxford University Hospitals NHS Foundation Trust, a tertiary centre in England, UK. The study was approved by the South Central—Oxford A Research Ethics Committee (ref:10/H0604/51) and is registered with the National Institute for Health Research (NIHR) UK portfolio (10776). Buffy coats from healthy donors were obtained from Sanquin Blood Supply, Amsterdam, Netherlands, and approved by the Medical Ethics Committee of Sanquin, Amsterdam, Netherlands. All participants provided informed written consent, human samples were handled in accordance with The Human Tissue Act, and the study was conducted in accordance with the Declaration of Helsinki.

A diagnosis of IgG4-RD was made using Comprehensive Diagnostic Criteria [26,27] and ACR/ELAR classification criteria for IgG4-RD [28]. A diagnosis of PSC was made using the British Society of Gastroenterology and the European Association for the Study of the Liver guidelines [9,29]. Serum IgG and IgG4 levels were measured by nephelometry.

### 2.2. Lymphocyte Isolation and CD19+ Cell Separation

Peripheral blood was taken in sodium heparin tubes from 14 study participants with active IgG4-RD, 8 patients with PSC-hIgG4 and 8 healthy donors (4 from Buffy Coats).

Published data on healthy subjects investigating the surface expression of these receptors on B cells found statistically significant differences between 8 and 10 individuals per group [4]. Therefore, we chose a similar sample size for this exploratory investigation.

Peripheral blood mononuclear cells (PBMCs) were isolated using Lymphoprep density gradient centrifugation (STEMCELL Technologies Vancouver, BC, Canada, catalogue number 07851) and stored frozen in liquid nitrogen. CD19+ B cells were isolated from thawed PBMCs using magnetic column separation with Dynabeads CD19 Pan B and DETACHaBEAD CD19, as per the manufacturer’s instructions (Invitrogen, Thermo Fisher Scientific, Boston, MA, USA catalogue number 11143D and 12506D).

### 2.3. Surface Staining and Flow Cytometry

For phenotypic analysis of B cells, isolated CD19+ cells were placed in 96 well plates (1 million cells per well) and stained with DAPI and a cocktail of fluorochrome-conjugated antibodies including CR1, CR2, FcεRII, FcγRIIb, CXCR3, CXCR4, CXCR5, CCR5, CCR6 and CCR7 IgG1 and IgG4 (Table 1). Cells were acquired using an LSR II flow cytometer (BD Biosciences) and analysed using FlowJo version 10.9 (TreeStar, Ashland, OR, USA).

### 2.4. Statistical Analysis

Statistical tests were non-parametric, as our data points were from human subjects, where data are assumed to be non-normally distributed. To analyse unpaired data, individual data points were grouped by disease state (healthy, PSC-hIgG4 and IgG4-PB) and the Kruskal–Wallis test with Dunn’s multiple comparisons was applied to make statistical comparisons between these groups. To analyse paired data, individual data points derived from the same individual sample were statistically compared by applying the Friedman test with Dunn’s multiple comparisons. Outliers were included in the statistical analysis. Statistical significance was defined as a *p*-value of under 0.05.

## 3. Results

### 3.1. Cohort Characteristics

Patients in the IgG4-PB and PSC-hIgG4 groups had similar age, gender and serum IgG levels. The median IgG4 level was higher in patients with IgG4-PB (Table 2). All patients with IgG4-PB had active disease at the initial presentation or disease relapse. Only one patient was on any immunosuppressive therapy at the time of sample acquisition (low maintenance dose of 5 mg once a day oral prednisolone).

### 3.2. Complement Receptors Are Not Differentially Expressed on B Cells in IgG4-PB

**Figure 1:**
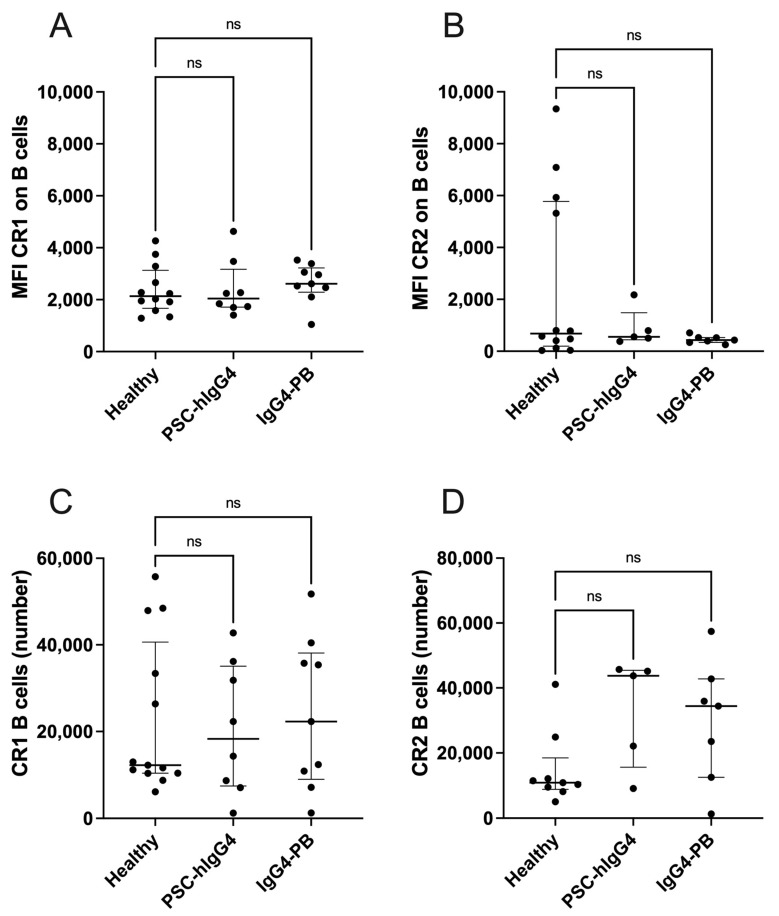
Expression of complement receptors 1 and 2 on ex vivo Total B cells from healthy volunteers compared with patients with PSC-high IgG4 (PSC-hIgG4) and patients with IgG4-pancreatobiliary disease (IgG4-PB) measured by flow cytometry. Median fluorescence intensity (MFI) of (**A**) complement receptor 1 (CR1, CD35) (**B**) and complement receptor 2 (CR2, CD21) on total B cells and total number of B cells positive for (**C**) CR1 and (**D**) CR2. A Kruskal–Wallis test with Dunn’s multiple comparisons is used to compare two or more unpaired groups. Median and interquartile ranges are shown. ns = not significant.

The surface expression of complement receptor 1 (CR1, CD35) and complement receptor 2 (CR2, CD21) was investigated on circulating B cells. There were no differences in expression of CR1 (Figure 1A) nor CR2 (Figure 1B) on circulating B cells nor in total number of B cells expressing these receptors (Figure 1C,D) between IgG4-PB, PSC-hIgG4 and healthy controls.

### 3.3. Complement Receptor 2 Is Differentially Expressed on IgG1+ and IgG4+ B Cells in IgG4-PB

**Figure 2:**
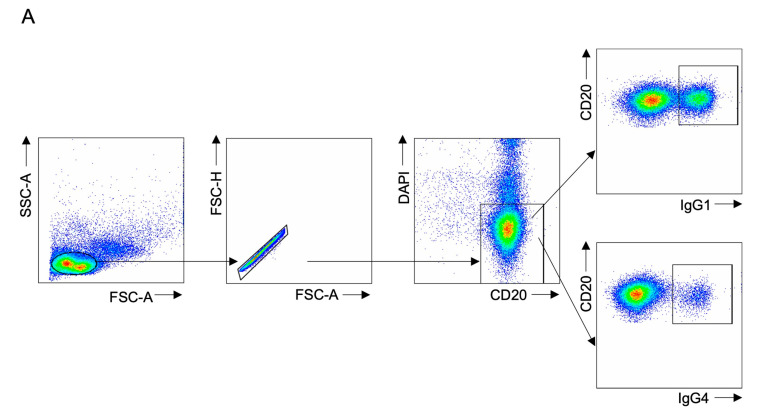

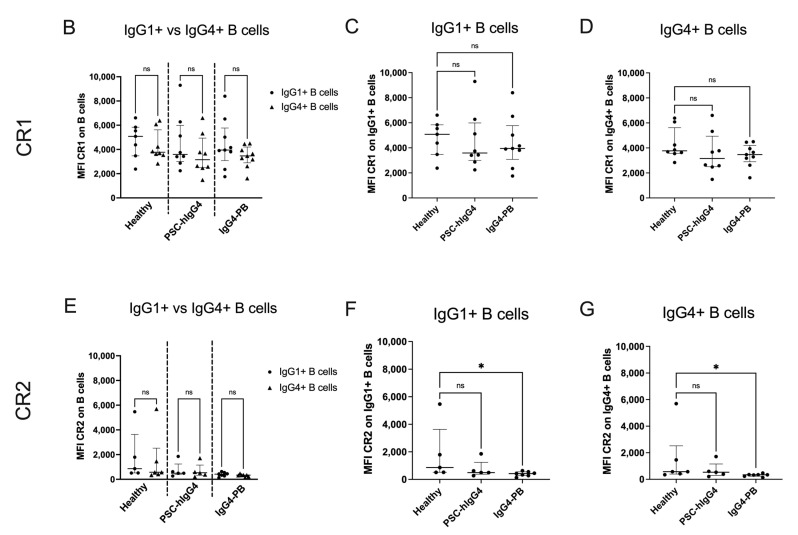
Expression of complement receptors 1 and 2 on ex vivo IgG1+ and IgG4+ B cells from healthy volunteers compared with patients with PSC-high IgG4 (PSC-hIgG4) and patients with IgG4-related pancreatobiliary disease (IgG4-PB) measured by flow cytometry. (**A**) Gating strategy to identify IgG1+ and IgG4+ B cells with each dot representing a single cell (colored blue to red, indicating increasing intensity). Populations gated from left to right; lymphocyte population by forward scatter area (FSC-A) and side scatter area (SSC-A), singlet cells population by FSC-A and forward scatter-height (FSC-H), live CD20 B cell population by DAPI negative CD20 positive, IgG1+ and IgG4+ B cell population by IgG1 (upper box) or IgG4 (lower box) positivity. Median fluorescence intensity (MFI) of complement receptor 1 (CR1, CD35) on (**B**) IgG1+ compared to IgG4+ B cells (**C**) IgG1+ B cells and (**D**) IgG4+ B cells and complement receptor 2 (CR2, CD21) on (**E**) IgG1+ compared to IgG4+ B cells, (**F**) IgG1+ B cells and (**G**) IgG4+ B cells. A Kruskal–Wallis test with Dunn’s multiple comparisons is used to compare two or more unpaired groups. Median and interquartile ranges are shown. * *p* < 0.05, ns = not significant.

The surface expression CR1 and CR2 was investigated on IgG1+ and IgG4+ B cells (gating strategy Figure 2A). There were no differences in CR1 expression between IgG1+ or IgG4+ B cells in healthy or disease states IgG4-PB or PSC-hIgG4 (Figure 2B–D).

CR2 expression was not different on IgG1+ compared to IgG4+ B cells in healthy or disease states (Figure 2E). In patients with IgG4-PB compared to healthy volunteers, CR2 expression was lower on IgG1+ B cells (MFI 416 (275–552) vs. 865 (515–3631), *p* = 0.0408, Figure 2F) and IgG4+ B cells (MFI 337 (231–353) vs. 571 (398–2521), *p* = 0.0303, Figure 2G). Similar differences were not observed in PSC-hIgG4.

### 3.4. Fc Gamma Receptor 2b Expression Is Reduced on B Cells in IgG4-PB

**Figure 3:**
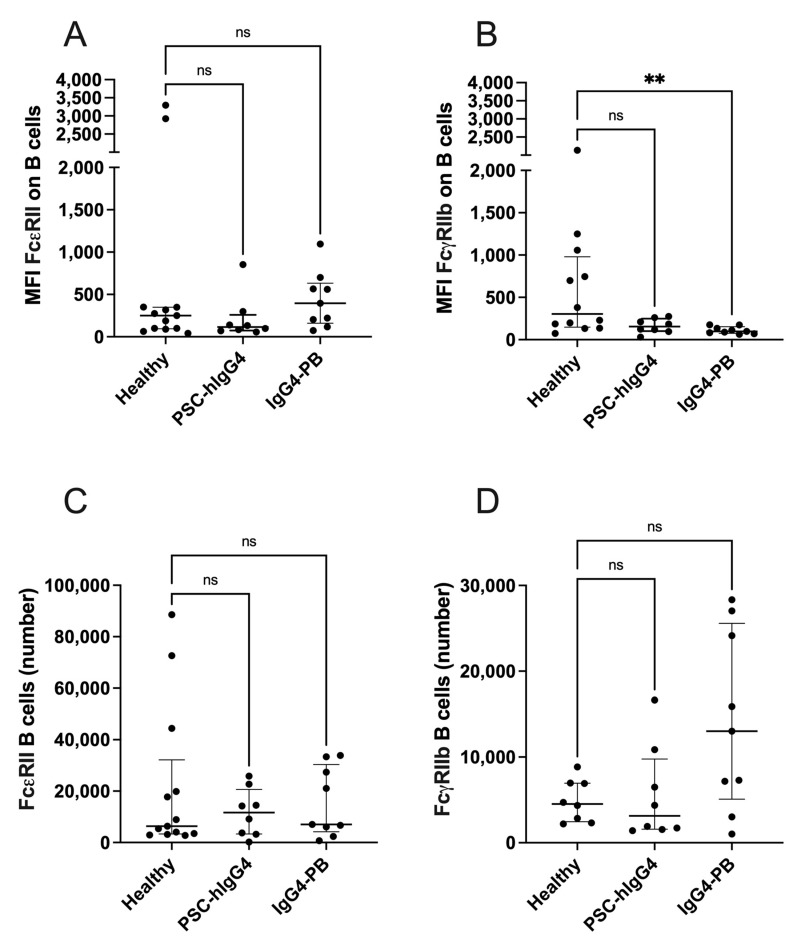
Expression of Fc receptors on ex vivo Total B cells from healthy volunteers compared with patients with PSC-high IgG4 (PSC-hIgG4) and patients with IgG4-related pancreatobiliary disease (IgG4-PB) measured by flow cytometry. Median fluorescence intensity (MFI) of (**A**) Fc epsilon receptor 2 (FcεRII, CD23) (**B**) and Fc gamma receptor 2b (FcγRIIb, CD32) on total B cells and the total number of B cells positive for (**C**) FcεRII and (**D**) FcγRIIb. The Kruskal–Wallis test with Dunn’s multiple comparisons is used to compare two or more unpaired groups. Median and interquartile ranges are shown. ** *p* < 0.01, ns = not significant.

The surface expression of Fc epsilon receptor 2 (FcεRII, CD23) and Fc gamma receptor 2b (FcγRIIb, CD32) was investigated on circulating B cells. There was no difference in the expression of FcεRII (Figure 3A), but FcγRIIb expression was significantly decreased in B cells from patients with IgG-PB compared to healthy controls (304 (149–979) vs. 98 (76–153), *p* = 0.0027, Figure 3B). There were no differences in total number of B cells expressing either receptor (Figure 3C,D). There was no association between serum IgE levels and B cell expression of FcεRII, nor serum IgG levels and B cell expression of FcγRIIb. 

### 3.5. Fc Receptors Are Differentially Expressed on IgG1+ and IgG4+ B Cells in IgG4-PB and PSC-hIgG4

**Figure 4:**
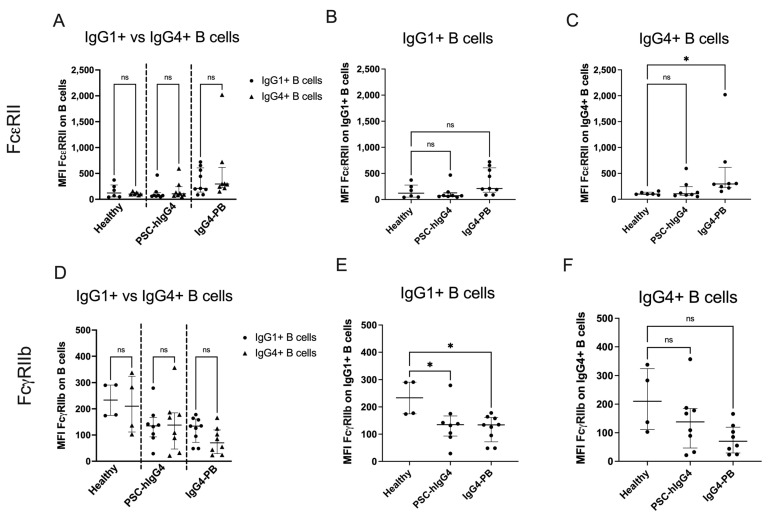
Expression of Fc receptors on ex vivo IgG1+ and IgG4+ B cells from healthy volunteers compared with patients with PSC-high IgG4 (PSC-hIgG4) and patients with IgG4-related pancreatobiliary disease (IgG4-PB) measured by flow cytometry. Median fluorescence intensity (MFI) of Fc epsilon receptor 2 (FcεRII, CD23) on (**A**) IgG1+ compared to IgG4+ B cells, (**B**) IgG1+ B cells and (**C**) IgG4+ B cells and Fc gamma receptor 2b (FcγRIIb, CD32) on (**D**) IgG1+ compared to IgG4+ B cells, (**E**) IgG1+ B cells and (**F**) IgG4+ B cells. A Kruskal–Wallis test with Dunn’s multiple comparisons is used to compare two or more unpaired groups. Median and interquartile ranges are shown. * *p* < 0.05, ns = not significant.

The surface expression of FcεRII and FcγRIIb was investigated on IgG1+ and IgG4+ B cells. There was no difference in expression of FcεRII between IgG1 and IgG4+ B cells, nor on IgG1+ B cells (Figure 4A,B). FcεRII expression was higher on IgG4+ B cells in patients with IgG4-PB compared to healthy volunteers (MFI 296 (225–617) vs. 100 (92–138), *p*= 0.0145, Figure 4C). There was no similar difference in expression in PSC-hIgG4. FcγRIIb expression was no different between IgG1+ compared to IgG4+ B cells (Figure 4D) but was lower on IgG1+ B cells in patients with IgG4-PB and PSC-hIgG4 compared to healthy volunteers (134 (72–161) vs. 234 (175–291), *p* = 0.0262 and 135 (93–167) vs. 234 (175–291), *p* = 0.0464, Figure 2E). There was no difference in expression on IgG4+ B cells (Figure 4F).

### 3.6. CXCR4 Expression Is Increased on B Cells in IgG4-PB

**Figure 5:**
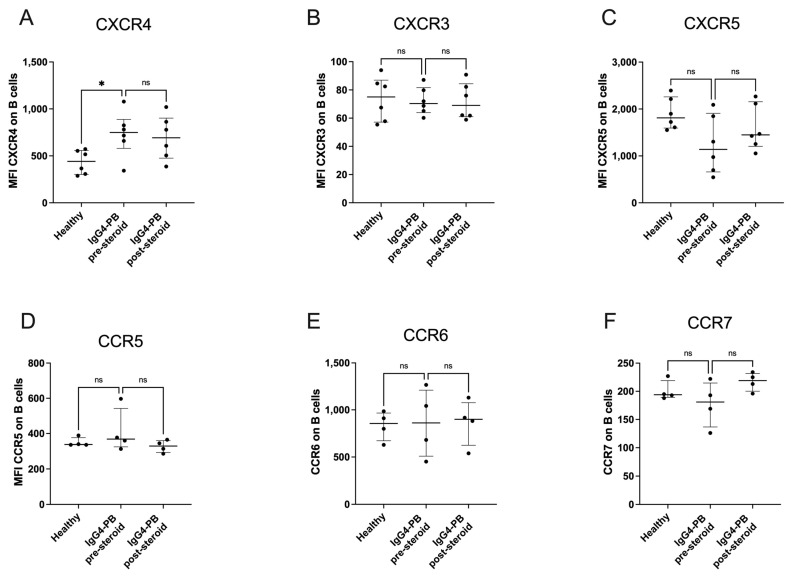
Expression of Chemokine receptors on ex vivo B cells from healthy volunteers compared with patients with IgG4-related pancreatobiliary disease (IgG4-PB) pre or post-steroid. Median fluorescence intensity (MFI) of (**A**) CXCR4, (**B**) CXCR3, (**C**) CXCR5, (**D**) CCR5, (**E**) CCR6 and (**F**) CCR7. A Kruskal–Wallis test with Dunn’s multiple comparisons is used to compare two or more unpaired groups. Median and interquartile ranges are shown. * *p* < 0.05, ns = not significant.

The surface expression of chemokine receptors was investigated on B cells in healthy controls and patients with IgG4-PB pre- and post-steroid therapy. All patients treated with steroids responded to therapy. CXCR4 expression was increased on B cells from patients with IgG4-PB pre-steroid therapy compared to healthy controls (442 (302–558) vs. 749 (580–888), *p* = 0.0411, Figure 5A). There were no differences in expression of CXCR3, CXCR5, CCR5, CCR6 and CR7 (Figure 5B–F).

## 4. Discussion

This is the first study to investigate the surface expression of complement, Fc and chemokine receptor expression on B cells in IgG4-PB and PSC-hIgG4. Our data show important differences in receptor expression between healthy volunteers and patients with PSC-hIgG4 or IgG4-PB and between IgG1+ and IgG4+ B cells, pointing to potential mechanisms through which these B cells exert effector functions in health and targets for immunomodulatory therapy in disease.

Complement receptors CR1 and CR2 are both expressed in human B cells and have several functions in engagement with antigen opsonised with C3-derived complement fragments [30,31]. Traditionally described as a B cell activating co-receptor, CR2 co-ligates with the BCR on binding complement opsonised antigen in conditions of low antigen concentration [32]. Although this leads to B cell activation in mice [33], in humans, it inhibits B cell proliferation, antibody production and differentiation into plasmablasts [32,34]. CR2 also plays a role in enhancing antigen presentation by B cells by promoting fixation and internalisation of antigen-complement complexes [35]. In previous work [4], we identified lower CR2 on IgG4+ compared to IgG1+ B cells in healthy volunteers, which may not have been observed in this study due to the small sample size. In IgG4-PB, compared to healthy volunteers, we find CR2 expression on IgG1+ and IgG4+ B cells is reduced. This suggests that both IgG1+ and IgG4+ B cells are less sensitive to complement-mediated inhibition via CR2 in IgG4-PB and are less likely to act as antigen-presenting cells to other cells of the adaptive immune system. Complement consumption is a feature of IgG4-RD, especially but not exclusively in those with IgG4-related tubulointerstitial nephritis [36,37,38,39]. Complement inhibitor Compstatin (Pegcetacoplan) targeting C3 might be a therapeutic strategy in these patients [40,41]. Further investigation is required to understand whether differential B cell expression of CR2 is associated with complement levels and disease activity.

Fc receptors bind the Fc portion of immunoglobulins, leading to several downstream immunomodulatory effects [42]. FcεRII expressed on B cells is a low-affinity receptor for IgE and preferentially binds IgE-allergen complexes, providing an alternative pathway to mast-cell sensitisation and degranulation [43]. On binding, FcεRII reduces B cell proliferation, differentiation and IgE synthesis [44,45,46]. We find FcεRII is increased on IgG4+ B cells in patients with IgG4-PB compared to healthy volunteers. This might be the result of shared induction factors such as interleukin(IL) -4 and IL-13 inducing both FcεRII expression and IgG4+ class switch [47,48] or a negative feedback response in the subset of IgG4-RD patients with raised IgE levels [49,50]. However, we found no correlation between serum IgE levels and B cell expression of FcεRII.

FcγRIIb is a low-affinity receptor for IgG, which, on co-engagement with the B cell receptor (BCR), leads to suppression of antibody secretion, acting as an antibody checkpoint to maintain B cell anergy [51,52]. We find the expression of FcγRIIb is reduced on IgG1+ B cells from patients with either PSC-hIgG4 or IgG4-PB compared to healthy volunteers. This suggests that in PSC-hIgG4 and IgG4-PB, anergic IgG+ B cells may be less susceptible to FcγRIIb-mediated inhibition and apoptosis, allowing them to proliferate and differentiate into antibody-secreting cells. In previous work [4], we identified higher FcεRII expression on IgG4+ compared to IgG1+ B cells in healthy volunteers (confirmed in an unpublished proteomics analysis; TR), which may have gone undetected in the present study due to the small study size. Targeting FcγRIIb is a therapeutic strategy in IgG4-RD under investigation. Obexelimab (Xmab5871) is a humanised anti-CD19 antibody with an Fc portion that binds FcγRIIb. A recent phase II trial (NCT02725476) has reported good safety and efficacy [53], and a multicentre placebo-controlled phase III trial is currently recruiting (NCT05662241, INDIGO Study).

Chemokine receptors bind secreted proteins of chemokine families, including CC and CXC [54]. Migration of B cells and plasma cells to and within germinal centres and secondary lymphoid organs is mediated through CXCR4-CXCL12 signalling [55,56]. We find increased expression of CXCR4 on B cells in IgG4-PB pre-steroid treatment, suggesting their increased migration to B cell follicles might be mediated through this pathway. CXCR4 expression reduces after successful therapy with steroids, suggesting its expression on B cells may have a role in disease pathogenesis. Circulating levels of the CXCR4 ligand CXCL12 were not measured, which will be important in interpreting this finding and should be measured in future studies.

This study has limitations. As a single-centre descriptive cohort, interpretation is limited by the small sample size and cannot be generalised to patients without pancreatobiliary manifestations of IgG4-RD. Replication in a larger cohort, including different points in the disease course, will be necessary to understand how differences in B cell phenotype contribute to disease pathogenesis. Receptor expression should be investigated before and after treatment in order to assess whether the expression is associated with treatment response.

Secondly, this analysis was limited to a small number of complement, Fc and chemokine receptors. The circulating levels of ligands such as complement fragments and chemokines were not measured, which could affect receptor expression. To address this, future studies should include simultaneous analysis of B cell expression of complement, Fc and chemokine receptors using multiparameter flow cytometry alongside circulating concentrations of all receptor ligands. The receptors investigated should be further expanded to include additional Fc receptors associated with B cell regulation, including FcγRI, FcγRIII and the Fc alpha receptor I (FcαRI).

Finally, B cell receptor expression in the peripheral blood may not represent the changes that occur within affected organs. It will be important to assess the expression of Fc, complement and chemokine receptors on B cells within the tissue of active IgG4-RD lesions and biliary ducts affected by PSC to further understand how they might contribute to organ inflammation and fibrosis.

In conclusion, this is the first study to investigate complement and Fc receptor expression on B cells in IgG4-PB and PSC-hIgG4. In IgG4-PB, B cells have decreased CR2 and FcγRIIb expression and increased FcεRII expression, suggesting they have altered sensitivity to complement, IgG-mediated inhibition and sensitisation by IgE which may promote the relative expansion of IgG4+ B cells in this disease. Future studies should investigate the functional consequences of the divergent B cell receptor expression and determine whether it is associated with clinical outcomes.

## Figures and Tables

**Table 1:** Surface staining antibody cocktail.

Antibody	Fluorochrome	Supplier	Clone	Cat No.
CR1/CD35	PE	BD Biosciences, Franklin Lakes, NJ, USA	E11	559872
CR2/CD21	APC	BD Biosciences, Franklin Lakes, NJ, USA	B-ly4	599867
FcεRII/CD23	PE	BD Biosciences, Franklin Lakes, NJ, USA	M-L233	555711
FcγRIIb/CD32	APC	BD Biosciences, Franklin Lakes, NJ, USA	FLI8.26	559769
CXCR3/CD183	PerCPCy5.5	BD Biosciences, Franklin Lakes, NJ, USA	IC6/CXCR3	560832
CXCR4/CD184	PE	BD Biosciences, Franklin Lakes, NJ, USA	12G5	561733
CXCR5/CD185	PECy7	eBioscience, San Diego, CA, USA	MU5UBEE	25-9185
CCR5	BV605	BD Biosciences, Franklin Lakes, NJ, USA	2D7/CCR5	563379
CCR6	PECy7	BD Biosciences, Franklin Lakes, NJ, USA	11A9	560620
CCR7	PE	R&D Systems, Minneapolis, MN, USA	150503	FAB197P-025
CD20	VB	Miltenyi Biotech, Bergisch Gladback, Germany	LT20	130-094-167
IgG4	APC	Sanquin Reagents, Amstedam, Netherlands	MH164.1	-
IgG1	FITC	Sanquin Reagents, Amstedam, Netherlands	MH161-1	-
DAPI	-	BioLedgend, San Diego, CA, USA	-	422801

**Table 2:** Baseline cohort characteristics.

	IgG4-PB	PSC-hIgG4
**Demographics**		
Gender male (*n*, %)	8 (57)	5 (63)
Age (median, range)	67 (39–82)	56 (36–76)
**Serum immunoglobulins**		
IgG (g/L, median, range)	16.4 (8.2- 23.7)	16.3 (12.1–35.8)
IgG4 (g/L, median, range)	5.9 (0.4–19.6)	2.0 (1.5–8.0)
IgE (kUL, median, range)	269 (11.4–1711)	138 (11.3–4452)
**Clinical status**		
Initial presentation	9 (64)	-
Relapse	5 (36)	
**Immunosuppression**		
No therapy (*n*, %)	13 (93)	-
Low-dose steroids (*n*, %)	1 (7)	

## Data Availability

Original data files will be made available on request.
